# The role of indigenous knowledge in disaster risk reduction and climate change adaptation in Chikwawa, Malawi

**DOI:** 10.4102/jamba.v16i2.1810

**Published:** 2024-11-14

**Authors:** Isaac K. Mwalwimba, Mtafu Manda, Cosmo Ngongondo

**Affiliations:** 1 Department of Physics and Biochemical Sciences, School of Science and Technology, Malawi University of Business and Applied Sciences, Blantyre, Malawi; 2 Department of the Built Environment, Faculty of Environmental Sciences, Mzuzu University, Luwinga Mzuzu, Malawi; 3 Department of Geography, Earth Sciences and Environment, School of Natural and Applied Sciences, University of Malawi, Zomba, Malawi

**Keywords:** adaptation, climate change, indigenous knowledge, variability, vulnerability

## Abstract

The role of indigenous knowledge in disaster risk reduction (DRR) and climate change adaptation cannot be underestimated. It acts as a preparedness and response tool to climate change-related impacts such as floods, droughts and strong winds. However, inadequate studies about indigenous knowledge in Malawi is a major challenge when dealing with extreme climatic conditions. Learning from indigenous knowledge systems, by investigating first what local communities know and have, can improve the understanding of local conditions and can provide a productive context for activities designed to help communities reduce vulnerability to climate change impacts. This paper assessed the role of indigenous knowledge systems in DRR and climate change variability and adaptation strategies in Chikwawa district. The study used a participatory research approach involving interactive research methods such as focus group discussions (FGDs), key informant interviews and participant observations. Data from key informants and FGDs were analysed thematically. The study revealed various indigenous knowledge which communities in the Chikwawa district use to respond to climate-related impacts such as floods. Some of these include hippopotamus relocating from the river to the village, extreme hissing of pythons in nearby forests, buffaloes and zebras wreaking havoc in the villages and crocodiles flocking to the village.

**Contribution:**

The study concludes that indigenous knowledge provides the basis for problem-solving approaches for local communities, hence, a need to document it at a wider scale.

## Introduction

Indigenous knowledge (IK) is traced through the ethnographic study of different scholars that have built up interest in it (Gigler 2005). In the study of IK, several studies have been carried out in order to reconstruct, understand and document IK. Langill and Landon (1998) describe IK as the process that involves construction of the knowledge which is done through experience and observation designed to operate as the process in generation as it passes from one generation to another. Kiran (2015) states that the construction and development of IK is carried out in the way that it has to fulfil the requirements of the community.

Indigenous knowledge is considered to be a body of information and skills existing within or acquired by local people over a period of time through the accumulation of experiences, society–nature relationships, community practices and institutions, and by passing it down through generations (Hakim 2012). Indigenous knowledge is important and local people hold a wealth of information and experience that represent a significant resource in the implementation of adaptation strategies in disaster risk reduction (DRR) and climate change-related shocks. This knowledge has emerged as a unique global approach for exploring needs and priorities in climate change variability. According to Hiwasaki et al. (2014), IK has been receiving a lot of attention since the early 1990s in the field of climate change. Mwaura (2008) states that IK has made local communities live in harmony with their environment for long periods because they can practise their own coping mechanisms and preparedness measures.

Indigenous knowledge in Malawi is culturally specific and complements existing early warnings to climate change-related disasters (DODMA 2013). However, according to Mwalwimba (2017), little has been done to understand, acknowledge and document the role of IK systems in Malawi. Iloka (2016) agrees that there is also the problem of documentation of IK because African IK tends to be embedded in people’s memories. This paper therefore aims at identifying and documenting IK systems used in DRR and climate change adaptation in a Malawian setting as well as identifying roles of such IK in DRR and climate change adaptation strategies.

## Centralisation of indigenous knowledge

The definition of IK presents various approaches, perspectives and understanding that people have on it. The term IK presents vexing philosophical arguments because the term is understood based on the purpose that it serves in a particular study (Kiran 2015).

Indigenous knowledge is defined as the organisation of knowledge constructed by group of people through generation of living in close contact with nature (Johnson 1998; Langill & Landon 1998; Ngulube 2002). This simply means that indigenous people have capability to build up their knowledge in places they live. That knowledge is helpful in coping up with challenges that arise in their natural settings. Indigenous people pass knowledge from one generation to the other. Because knowledge is constructed in localities, it becomes part and parcel of the community in which it was generated. Ngulube (2002) argued that defining IK in that way simply shows understanding of the knowledge according to where it was generated. Lwoga, Ngulube and Stilwell (2011) also suggested that IK acts as the solution to the problems that arise in the environment. Such problems may include those that arise in agriculture, natural resource management, education and many more.

However, Flavier (1995) defines IK as ‘the local knowledge that is unique to a given culture or society. It contrasts with international knowledge system generated by universities, research institutions and private firms’. Indigenous knowledge in this case is considered as the knowledge generated within a small community. The definition also shows that there is uniformity of such knowledge in that community. This indicates that the knowledge is developed and owned within a certain locality. In addition to that, IK is also defined by comparing it with international knowledge. The definition indicates that while IK originated in communities, international knowledge is developed by institutions and utilised by nations. This approach of understanding IK is considered as an integrated approach because it looks at relationships of different elements which can assist communities to withstand hazardous events.

Koritha (2007) and Kiran (2015) also defined IK as the long-standing information that also encompasses practice, tradition and wisdom owned by a group of people in a well-defined community. That is to say, the traditions, wisdom and practices of the community that are taught to the young generation to survive. The knowledge, in this case, is considered as the important tool for the community to cope up with challenges for the existence of the people (Koritha 2007). Another important aspect that the definition provides is the term, ‘long standing information’. This term simply indicates that the IK was established way back, and it tends to pass different period and is made up of things that are established in order to achieve an intended goal (Camacho et al. 2016). Generally speaking, the establishment of IK has been there for a long period of time and seems to be helpful in communities it was generated.

The study conducted by Nuffic and United Nations Educational Scientific and Cultural Organization (UNESCO) (2002) defined IK in comparison with modern scientific knowledge. It defined IK as the set of knowledge and skills that was developed outside the formal education system. Formal education system, in this case, describes the education system followed by various institutions such as primary schools, secondary schools, colleges, among others, which is well organised and follows a particular instruction (MOE 2006). The aim of providing formal education is to provide skills and modern knowledge to the people involved in it. Nuffic and UNESCO (2002) continue to highlight that IK is attached to culture and to a particular local community, and that it is provided by the community members. The knowledge is distributed uniformly to members through traditional media within the community rather than institutions.

Indigenous knowledge is also referred to as the societal norms, values, beliefs and cosmovisions that are guided through human interactions in any community or locality (Subramanian & Pisupati 2010). This implies that IK is gathered through community norms, values, beliefs and cosmovision that try to sustain indigenous people’s livelihood. The existence of the community in this case shows that there is a need for the organisation of knowledge, the knowledge as seen in norms, values and beliefs that the community has. The layout of IK in community provides the good basis for the formation of good community. The knowledge tends to direct various interactions that exist within the community.

Other scholars define IK in a more sophisticated way. For instance, Kiran (2015) understands IK as the understanding and interpretations of features from both plants and animals, which have been constructed and developed within the culture of the community and/or society. Culture in this context includes the way of life of people in terms of natural resource use, language, rituals and spirituality. The definition simply shows how IK is constructed and developed in the community. Kiran (2015) shows that IK involves understanding of a particular element of the community, for example, environment. It also interprets various environmental themes thereby giving out meanings that become part and parcel of the culture.

The above-stated definitions of IK show that different scholars define it differently. However, their definitions have similar approach in the way that, all definitions indicate that it is developed in local communities. The knowledge as the information tends to pass from one generation to another within the community. In addition to that, IK has salient features which differentiate it from another knowledge (Diale & Fritz 2007).

This study, however, understands IK as the knowledge that was developed by the people in their communities in past. The knowledge is capable of managing natural resources including forests available in that particular community (Sirima 2015). The knowledge is shown in various practices which the community follow. Indigenous knowledge tends to pass from one generation to the next and tries to adapt to changes that occur in the environment (Sirima 2015:110). This implies that the knowledge is established in the community and provides solutions to the problems that affect the community. The knowledge is developed in every period of time including past, present and future, and is the agent of change.

## Features of indigenous knowledge

Literature provides various categorisation of IK. Acharya and Predeep (2016) classified IK existing in communities into ‘ecological’ and ‘meteorological’ aspects. While ecological aspects relate to non-human behaviour, phenomena and patterns, the meteorological aspects relate to wind movements, cloud and rain patterns. Woytek and Gorjestani (1998) and Kiran (2015) further show five features of IK, as discussed next, that depict and help to provide basic understanding of IK.

### Indigenous knowledge is local

This simply means that IK is rooted to specific communities and connected to general cultural traditions. The knowledge is also traced through indigenous people who live in that particular locality long years ago (Nyumba 2006). The knowledge is usually expressed in local language, which enhances sharing of information within a specific community. The knowledge is generated by them in order to sustain their basic life needs. It has been noted that indigenous people through their local knowledge were good in terms of resource allocation (Kajembe 1994; Schmidt 2020; Sirima 2015). In terms of distribution patterns of IK, it spreads uniformly in that particular local community. The knowledge is practised within a defined territory and has little or no influence on other communities.

### Indigenous knowledge is community-specific

Indigenous knowledge is a set of experiences generated by people living in a specific community (Wigrup 2005). This entails that IK is developed based on experiences of people in their communities. The indigenous people pass through periods of time in life. Through those periods of time, people experience a lot of changes which are usually enhanced because of changes in the environmental conditions. When such changes or problems arise in a community or a country, people come up with ways of facilitating solutions (Nyumba 2006). The ways which provide solutions that the communities adopt are those that are attached to the community. They become an integral part of the community; this encourages the community to pass that information from one generation to another. Woytek and Gorjestani (1998) indicated in their study that the knowledge that is practised is community-specific and contextual. This simply shows that communities have different approaches towards a particular problem. The ways of dealing with the problem may differ because of economic status. Nuffic and UNESCO (2002) highlighted that IK plays a critical role in the lives of the poor. For example, coronavirus disease 2019 (COVID-19) disturbed the entire world. As a way of dealing with COVID-19, face masks were used by people as a preventive measure (WHO 2019). Because of the cost of the industrial mask, people in various communities ended up making their own masks. The knowledge of making simple masks helped poor communities to cope with the problem of COVID-19 using limited resources available. This clearly shows that people develop their knowledge based on the experiences in their local communities. In the similar vein, Grenier (1998) suggested that IK system is holistic and as such new knowledge is continuously added to it. This is on account of the dynamism of environment which is attributed to the constant changes that are occurring, necessitating the knowledge to align and evolve with the changing environment.

### Indigenous knowledge is tacit

Indigenous knowledge is considered to be tacit that is not easily explainable (Kiran 2015). Nyumba (2006) stated that the knowledge is transmitted by indigenous people. This prevents others who are not part of the community or those who do not share similar language, tradition and other cultural experiences, to understand it. The knowledge is developed in order to solve challenges affecting a specific community. The development of the knowledge depends on the community resources and its understanding by the people. Therefore, knowledge is complex in that it is based on the understanding of local people. This makes it difficult for other people to understand, who are not part of the community where it was developed.

### Indigenous knowledge is transmitted through imitation, demonstration or orally

Indigenous knowledge is passed from one generation to the other orally, or through imitation and demonstration (Kiran 2015; Wigrup 2005; World Bank 2002). For example, elders may teach young ones how the community manages forest through songs, rituals, folklore, among others. The knowledge is discovered by the indigenous people who do not know how to read and write. This influences the community in passing the knowledge using traditional mediums (Nuffic & UNESCO 2002). The knowledge is strictly attached to the community, where it is practised as their way of life. For instance, in Malawi, tribal groupings have different traditional practices that are used to teach young people about the different knowledge used by indigenous people in the past. This helps all members of the community to be aware of it and adopt it as a part of their life.

### Indigenous knowledge is experimental rather than theoretical

Indigenous knowledge involves practices that are developed through trial and error in order to be utilised as the way to solve challenges in the community (World Bank 2002). Indigenous knowledge is gathered through observations and experiences over a long time frame and tries to adapt to the dynamic environment and the local culture (Nuffic & UNESCO 2002; Nyumba 2006:3; Wigrup 2005). Nuffic and UNESCO (2002) added that the knowledge is developed by the community and it keeps on developing it. The knowledge is tested for its use and is adapted to the local culture and the changing environment and dynamics. This indicates that the knowledge is based on valid information that the community discover.

The above-stated features of IK enable researchers who are interested in the study of IK to have a better understanding of it. Based on the following definitions and features of IK, this research study followed similar tacit but with a different approach on how to define IK. This study took IK as the knowledge developed and practised in local communities by indigenous people in order to manage forest resources and pass it from one generation to another for the survival of people and other living things. Indigenous knowledge in this context is not embedded in modern knowledge, mainly because it has not been well-defined in the documents. The survival of IK depends on the community practices.

## Indigenous knowledge and knowledge systems

Indigenous knowledge possesses a distinct characteristic that differentiates it from other knowledge systems. This difference between IK and other knowledge systems has been brought up in various debates. Two schools of thought, one supports IK while the other tends to support scientific knowledge. The debate has created tension among people because each knowledge system wants to dominate the other. However, Langill and Landon (1998) and Kiran (2015) argued that it is better not to be involved in this debate but to engage with and use both knowledge systems complementarily to achieve the required development. Therefore, to have a deeper understanding of IK, this paper highlights some of the unique features that IK has when compared to other knowledge systems.

Richards (1980) and Brokensha, Warrent and Werner (1985) describe IK system as a closed system. The closed system is also known as an impermeable system because it does not allow other materials to enter into the system (Holden 2005). When the system does not interact with the external environment, it is difficult to be modified. Therefore, IK is said to be a closed system mainly because it is the knowledge of a particular locality or is propagated by a particular local community. Richards (1980) stated that IK is characterised by lack of awareness on the ways of depicting the world, from an open or permeable system. This means that IK does not allow modification of the systems to take place because people tend to hold their own values in the society. The study conducted by Richards (1980) indicated that, scientific knowledge is always subject to changes that may come depending on the perspectives of the people who adopt it in a particular period.

The organisation of knowledge is another area of difference between IK and scientific knowledge. Indigenous knowledge is said to be loosely organised and is associated with local people who have typical respect of their values (Thrupp 1989). The knowledge is said to be scattered because of the origin in which it was developed. The development of the knowledge focusses much on the needs and problems of people in their respective community. This implies that the knowledge is developed in order to solve the problems in a particular area, thereby satisfying the needs of the people in that community. However, modern scientific knowledge is said to be organised in a centralised way and it is mainly operated by the work of the governments. That is to say, modern scientific knowledge is merged together in the way that it encompasses the people in various communities. The scientific knowledge is governed by state actors and/or scientists who can adopt new knowledge systems rather than the people themselves. People who utilise this type of knowledge consider themselves to be superior to those who utilise IK. Generally speaking, IK is different from other knowledge systems in the way that its organisation and utilisation are done within a small community which is different from other knowledge systems.

Indigenous knowledge also differs from other knowledge systems according to the time period in which the knowledge was developed. Indigenous knowledge is seen as backward, static and a barrier to modernisation (Appleton et al. 1995). The word IK indicates knowledge developed in the past generated from indigenous people or ancestors. This led people to think that IK is for the people living in the past. Therefore, IK is considered static and that it cannot be modified. Based on the different perspectives that people have towards IK, it is considered as a barrier to development. Contrarily, scientific knowledge is considered as the modern knowledge for development (Appleton et al. 1995). Many people consider modern knowledge as the civilised and the best knowledge for development. Therefore, the perspectives that people have about indigenous and scientific knowledge provide differences that exist among them.

The contributions to development of the rural communities furthermore display the differences that exist between indigenous and scientific knowledge system (Tripathi & Bhattarya 2004). According to Wigrup (2005), IK plays a role in the management of resources in rural communities across the world. According to Brokensha et al. (1985), IK encourages participation in various communities. This is necessary when it comes to the development of the society as well as the whole nation. Participation of people in various developmental activities helps people to contribute their ideas. The ideas that people contribute help to structure the developmental activities thereby ensuring development in that particular community. Scientific knowledge systems have contributed little to the development of the rural communities across the world (Tripathi & Bhattarya 2004). This is as a result of the lack of participation and interest shown by the local communities. The scientific knowledge in local communities is viewed as the knowledge which tends to uproot the role that indigenous people have. This makes the local communities disinterested in scientific knowledge. Indigenous knowledge thus differs from other knowledge systems in terms of the contributions that it makes in rural development.

## Research methodology

The study used a participatory research approach. Data were collected using focus group discussions (FGDs), key informant interviews (KIIs) and observations. This study was carried out in Group Village Headman Kalima in Traditional Authority Maseya of Chikwawa district in the southern part of Malawi.

The target population comprised indigenous leaders (chiefs and elders), Area Civil Protection Committee (ACPC), Village Civil Protection Committee (VCPC), Village Development Committee (VDC), youth representatives, religious representatives and women representatives These groups were selected for various reasons as outlined in Table 1.

**TABLE 1:** Target population of the study.

Title	Reason or purpose
Village Headman	Involvement as a leader and his ideas in IK
Chairman of the VDC	Involvement as a leader and his ideas in IK
Village District Committee Member	Inolved as members of the village disaster risk management to provide ideas on IK
Member of the ACPC/VCPC	Involved because they are part of disaster risk management implementation
Religious representative (Pastor)	Provided ideas from the religious perspective
Women representatives	Input ideas from a feminine perspective in IK
Youth representatives	Input ideas in IK from youths’ perspective

ACPC, Area Civil Protection Committee; VCPC, Village Civil Protection Committee; IK, Indigenous knowledge; VDC, Village District Committee.

The study employed purposive sampling method for selecting 15 key informants (chiefs and elders), 2 youth representatives, 2 women representatives as well as 1 member of the Area Civil Protection Committee (ACPC) and 10 members of the Village Civil Protection Committee (VCPC).

According to the demographic characteristics of the participants, 53.1% were females and 46.9% were males. As for the age, majority of the participants (43.8%) were between the age of 51 and 60 years based on the assumption that the elderly are well informed in terms of local or IK. With reference to education, 40.6% of the participants affirmed that they know how to read and write, while 59.1% of the participants confirmed that they do not know how to read and write. The main economic activity in the area was farming as it was practised by 75.0% of the participants. One main result from this analysis is that low education of participants limits their understanding of the scientific knowledge provided by the Department of Climate Change and Meteorological Services (DCCMS).

Data from KIIs and FGDs were analysed thematically. The data were read and examined carefully to provide a detailed description of themes or patterns of cultural meaning. The data were classified based on the themes IK, DRR and climate change adaptations. Key themes which were analysed included demographic characteristics of the participants, kinds of IK, IK and scientific knowledge as well as IK in DRR and climate adaptation strategies.

### Ethical considerations

Ethical approval to conduct this study was obtained from the Mzuzu University Research Ethics Committee (No. MZUNIREC/DOR/21/37).

## Results and discussion

### Demographic characteristics of the participants

#### Indigenous knowledge identified

The analysis established various kinds of IK which were grouped according to Acharya and Predeep’s (2016) categorisation system. The system categorises IK which deals with non-human behaviour, phenomena and patterns, and those which deal with wind movements, cloud and rain patterns.

#### Ecological category

The ecological category deals with patterns relating to the behaviour of animals and plants. The analysis and our observations revealed various IK dealing with animals and plants which helps communities to respond positively to climate change shocks such as floods and droughts (Table 2).

**TABLE 2:** Ecological indigenous knowledge dealing with animals.

Indigenous knowledge	Possible hazard implication
Hippopotamus relocating from theriver to the villages	Too much rainfall with likely flooding
Frogs from Shire River flock to thevillages and start croaking	Too much rainfall resulting in flooding
Extreme hissing of pythons in nearby forests	Too much rainfall resulting in flooding
Buffaloes wreaking havoc in the village	Too much rainfall resulting in flooding
Ants emerging from the soil in large quantities	Too much rainfall resulting in flooding
Ground cricket digging up holesin gardens	Too much rainfall with likely flooding
Crocodiles flocking to the villages	Too much rainfall with likely flooding
Crabs shed off their shells	Implying little rainfall resulting in drought
Increased appearance of tsetse flies	Too much rainfall to be received
Increased appearance of millipedes	Too much rainfall to be received
Increase in Nang’omba birds	Too much rainfall to be received
Chickens flying up in the trees	High rainfall with likely flooding

#### Hippopotamus

The participants identified hippos as the first indicator of ecological IK because they respond to climate change-related events such as floods and droughts (Table 2). They indicated that the moving away of hippos (*Hippopotamus amphibius)* from Shire River to the dry lands helps them to know that there is going to be more rainfall which will likely cause flooding in that particular year. One of the participants highlighted that:

‘Hippos tend to wander around in the villages that are close to the river and in some cases, they wreak havoc. When the hippos behave in this way, it symbolises that, in that particular year, there is going to be much rainfall which will likely result into flooding.’ (54 years, male, religious leader in the community)

The participants further indicated that the distance covered by the hippos signifies how far the flood will reach. In this case, the participants acknowledged that they make sure that they keep enough food to help them during flooding time. They also indicated that this helps them to prepare evacuation points for their belongings including livestock. They also reported that this helps them to embark on the construction of canals and small dykes within their homes to reduce the amount of water reaching their homes.

#### Frogs (*Rana temporaria*)

The participants reported that when they see large flocking of frogs (*Rana temporaria*) from Shire River to nearby villages and they start croaking, it indicates that heavy rainfall will be received that year which will likely result in flooding. In this case, they prepare themselves for the impending flooding by making sure that they inform one another in the community to take stock of things such as food and resources so that they can be prepared for the emergency situation. They also indicated that they mobilise one another to conserve marginal areas especially through afforestation and catchment management programmes as one way of promoting and strengthening mitigation and adaptation strategies to climate change related impacts.

#### Pythons (*Pythonidae*)

The participants clearly indicated that the behaviour of pythons (*Pythonidae*) gives them an early warning of impending flooding. Hissing of pythons in the nearby forests and sugarcane plantations indicates heavy rains to be received. Such heavy rain fills up the Shire River and in turn causes flooding. One of the participants in the interviews explained:

‘During the past times, when this whole area was covered with trees, there used to be extreme hissing of pythons and in some cases some pythons produce similar sounds as those of cock crows. When that happens, we would know that rainy season has approached and we are going to receive so much rainfall. But now most of those forests are no more, and are replaced by the sugarcane plantations.’ (61 years, male, member of the disaster risk management committee)

Participants in the FGDs maintained that there is a need to encourage every member of society to engage in afforestation and environmental management in order to reverse the conditions of the environment. This will help to bring the pythons back to the area to serve as early warning system (EWS).

#### Crocodiles (*Crocodylinae*)

The participants reported that when they see crocodiles flocking to the nearby villages and wreaking havoc, it is an indication that in that year there will be more rains that will likely cause flooding. They also reported that other animals such as buffaloes (*Bubalus bubalis*) and zebras (*Equus quagga*) used to behave in a similar way. However, because of less forest cover, a good number of the remaining ones are kept in the nearby game reserves. Initially when the crocodiles, buffaloes and the zebras flocked to the villages, it meant that, in that year much rain will be received. One key informant reported that:

‘[*T*]his is a good indicator that we have been using for a long period of time to respond to climate change related events because most of us do not believe on the scientific ones as they are too much general.’ (59 years, female, member of the village development committee)

It was noted that communities use this knowledge as part of flood warning systems as it ensures time for evacuation of people and their belongings to safe areas.

#### Tsetse flies (*Glossina*)

The participants reported that an increase in the appearance of tsetse flies (*Glossina)*, owls (*Strigiformes*), millipedes (*Diplopoda*) and Nang’omba birds (Southern ground hornbill – *Bucorvus leadbeateri*) helps to predict the high amount of rainfall to be received in that particular year. Participants in the FGDs emphasised that:

‘[*T*]hese animals indicate that much rain is going to be received which will likely result into flooding.’ (54 years, male, participant of GVH Kalima, and member of VDC)

However, some key informants highlighted that because of changes in lifestyle and cultural dilution, the new generation is very resistant to learn these local ways. One of the key informants stated that:

‘[*D*]ue to cultural dilution, the young generation do not believe in indigenous knowledge as they consider them as old not fashion.’ (42 years, male, chairman of disaster risk management committee)

It was also observed that this problem is influenced because of the lack of inclusion of IK in decision-making processes in Malawi. This is because most of the young generation do not have any basis where they see IK being emphasised by policymakers or in policy documents or even being taught at school level. In order to improve on this, it was noted that there is a need to educate the young people about the importance of IK and encourage group discussions where such information should be shared among the youths.

#### Crabs (*Brachyura*)

The participants also reported that they know that when crabs (*Brachyura*) shed off their shells, then that particular year little rainfall is going to be received, and as a result, drought is imminent. They maintained that at this point they become aware that they should store food carefully to ensure the availability of food during dry season. Participants acknowledged that this helps them to participate in activities that cushion the effects of droughts such as early planting, planting drought-resistant crops and diversifying their farm activities. On the other hand, they reported that when they see ground crickets digging up holes in fields and destroying crops, they know that much rains will be experienced. This also helps them to predict about the onset of heavy rains and likelihood of flooding.

#### African red army ants

The prevalence of African red army ants (commonly known as linthumbwi) is a huge ecological indicator. When there is an increase in the occurrence of African red army ants, people know that there is going to be much rains which will be received, and in most cases, they result in flooding. One of the participants clarified:

‘When we see the African red army ants moving in masses, we know there is much rainfall. Such ants disturb people in their sleep by biting them, and in worse cases they attack and cut children hair while sleeping. When we see that we know that there is higher chance of rains and flooding which is bound to happen.’ (65 years, male, village community leader under Kalima)

The participants highlighted that the information helps them to avoid cultivating crops which do not require a lot of water. Participants also indicated that this information assists them to ask some local non-governmental organisations to support them with seedlings for early maturity crops. They said that these early maturity crops mature quickly such that during the time when floods happen, they sometimes have already harvested their crops, especially maize.

#### Plants

The participants reported some behavioural patterns of plants as foretelling what kind of conditions are expected, for example, whether the area is going to receive much rains or not (Table 3). Participants reported that local people know that an area will receive little rain which will likely result in drought when they see large quantities of mangoes and Nandolo being produced. Other behavioural plant patterns include the production of more fruits by ‘Mchulutsa and Gugu trees’. Such patterns, however, symbolise that an area will receive a lot of rainfall. Once noticing these, the participants reported that village chiefs during village meetings inform the communities about the threat of floods, and thereby strengthening awareness on floods and water courses for preparedness and response mechanism. However, it was observed that most of these plants are getting extinct because of changes in climate which has resulted in unpredictability of growth of these plants. It was therefore noted that the communities need to be encouraged to engage in afforestation so that these plants are conserved.

**TABLE 3:** Ecological indigenous knowledge dealing with plants.

Indigenous knowledge	Interpretation
Large quantities of mangoes produced	Less rains likely to result in drought
Large quantities of Nandolo produced	Less rains likely to result in drought
Mchulutsa trees producing much fruits	Too much rainfall is going to be received
Gugu (a fruit)	Too much rainfall and more likelihood of high yields
-	Portrays that there is going to be too much rainfall

#### Meteorological

Local knowledge of the movement and pattern of wind, clouds and rainfall in the study area also plays a significant role in forecasting either floods or heavy rains that could cause floods or to understand the extent of the damages caused by them (Table 4).

**TABLE 4:** Meteorological indigenous knowledge.

Indigenous knowledge	Interpretation
Change in wind direction in short period of time	Heavy rains will be experienced
Too much wind blowing	Little or no rains will be experienced
Too much heat	Drought to be experienced Much rainfall is going to be experienced
Appearance of stars (Nthanda)	Little or no rainfall to be received
Shady looking rock with tears or dry	If the rock looks with tears, it means in the year too much rainfall will be received, likely to result into flooding. If the rock looks dry, it means little rainfall likely to be drought condition.

#### Change of wind direction

Participants indicated that when the communities observe that the wind that was blowing from the south has changed suddenly to any of the three remaining directions (north, east and west), they know that heavy rains will be received. Heavy rains in this particular area fill up most of the Shire River tributaries and when the Shire River overflows, floods do occur. The change in the direction of wind helps them to prepare for an impending flooding. On the other hand, the participants reported that change in heavy blowing of winds signifies little or no rains to be received and such winds are usually destructive in nature.

#### Too much heat

The participants indicated that when too much heat is experienced between the months of August and October, it is an indication of heavy rainfall that is to be received in the rainy season. However, participants reported that when the heat persists, it is a sign that a dry spell will occur. These indicators help them to provide early warnings of impending flooding and/or drought. It also helps them to keep maize in reserves to be eaten during the time of inadequate harvest.

#### Appearance of stars

The participants also reported that when there is appearance of stars, it symbolises that the skies are clear, and clear skies mean that there are no rain-forming clouds, hence little or no rains to be received. It was observed that this assists communities to adopt new methods of farming such as mixed and inter cropping so that they can ensure food security in that particular year.

#### Rock with or without tears

The participants also reported that they have a big rock in their community which either produces tears or no tears. The participants highlighted that when the rock produces tears, it is an indicator that in that year the area is going to receive rainfall which is likely to cause flooding. On the other hand, if the rock produces no tears, it is an indication that in that year, the area is not going to receive rains, rather there will be drought.

### Indigenous knowledge versus scientific knowledge

Most participants who were interviewed preferred the use of IK to prepare for and predict about impeding hazards such as floods. It was observed that the major cause of the lack of trust in the scientific knowledge is the too much generalisation of early warning information provided by the DCCMS. One participant had this to say:

‘[*T*]he information we get from DCCMS is too general, it does not serve the purpose of warning us in this village.’ (45 years, female, member of the disaster risk management committee)

Most participants argued that the information from the DCCMS is not specific to what exactly is going to happen in a certain area. Furthermore, participants indicated that indeed the use of radio and television has widened the scope of information, but they maintained that they do not specifically inform the community at risk of flood hazards in their own locality. During FGDs, one participant said:

‘Most information we get from radio tend to be too general, and people lack trust on such because sometimes of the things that are mentioned do not happen.’ (43 years, male, community leader)

However, some participants highlighted that IK can complement science-based information systems. They maintained that the two systems can effectively work together only when they are combined. This is what one key informant had to say:

‘Scientific and local knowledge cannot work independently because both elements have their own shortfalls.’ (52 years, male, community member in group Kalima)

This key informant further maintained that the systems complement each other especially in the provision of early warning information on the impending hazards. The participants further maintained that IK systems must be incorporated in decision making especially in development projects because they help provide valuable information to communities about the risk of disasters. However, a major observation made is that very little has been done to integrate IK with scientific or modern systems in most policy documents in Malawi. Hence, this slow integration of IK systems combined with limited documentation of the same presents a gap in the work of DRR and climate change adaptation, because for many times they have assisted communities to prepare for and respond to natural hazards such as floods.

### Indigenous knowledge in disaster risk reduction and climate change adaptation

Our observations and analysis revealed that most IK practices assist communities to respond positively to climate change problems. Indigenous knowledge acts as EWS for communities to respond to impending hazards. From the analysis and observations, we find three roles of IK in DRR and climate change variability and adaptation in relation to the area where this study was carried out:

- Preparedness tool: Most people use IK to prepare for impending floods. It was observed that once the communities see these indicators, they get prepared, for example, by stocking up food and they move their livestock to higher grounds in readiness for the floods. Indigenous knowledge helps people to become aware of occurrence of floods or disasters so that they live in readiness.
- Foretelling tool: Indigenous knowledge helps people to predict the occurrence of disasters (floods or drought). Other people from this area believe that it foretells events that had not been foretold by science.
- Response tool: Indigenous knowledge helps people to respond to emergencies (increase response and awareness when disaster strikes). They are better able to develop their coping mechanisms to impending disasters through the use of IK systems. Communities are also able to identify their own means of disaster survival strategies with the help of IK systems.

### Implications and broader patterns of the study

The findings of this study posit the need to build on IK as a key strategy for EWSs in flood-prone areas of Malawi. This can help save lives, livelihoods and infrastructure, and support long-term DRR and climate change adaptation strategies. Strengthening of EWSs through IK would be a response to the United Nations Secretary-General’s (UNSG) call for ‘EWSs to reach everybody within the next 5 years (2023–2027)’. In the context of Malawi, if policies and actions are taken to build on IK, it would foster integration of IK and scientific knowledge systems by:

- strengthening communities’ access to communication, which will contribute to lower human vulnerability to climate risks;
- promoting interpretation of alert systems (IAS), because most participants revealed a lack of knowledge to interpret the levels of alerts associated with colours such as red, green and blue which have been highlighted in the National Contingency Plan (NCP), and being used as scientific systems in different catchments for flood prediction; and
- improving verification of effectiveness of EWS provided to the communities through forecast information and early weather messages.

Similarly, integration of IK and scientific knowledge systems can address several weaknesses and gaps that exist in Malawi. These include:

- limited incorporation of EWS in specific hazard early warning systems (SHEWS) and multi-hazard early-warning systems (MHEWS);
- a lack of people-centred EWSs (in terms of warning messages recognised and understood, and warning messages tailored to specific needs);
- lack of knowledge among communities affects the adoption of EWSs;
- limited technology for EWSs across all sectors. This should be strengthened to improve SHEWS and MHEWS;
- limited thematic hazard coverage and its cascading. These should be improved to effectively implement systems to reduce the risks; and
- capacity gaps in awareness and/or education, resilience building, preparedness infrastructure, monitoring or prediction, communication.

## Conclusion

This study has revealed that local communities use a lot of cultural beliefs to respond to climate change related impacts. Indigenous knowledge is considered an essential part of the development process of local communities, providing the basis for problem-solving approaches for local communities. Therefore, it is paramount for local communities to keep on learning and passing on such skills and information. The findings also reveal that building on the IK system existing in the study area can help in offering great prospects for effective integration of mitigation and adaption strategies in DRR and climate change related impacts. It is also important to strengthen dialogue and action in this crucial era of climate-related disasters by ensuring that:

- warning messages given for complex disasters are simple, clear and actionable with the inclusion of IK systems;
- multiple channels are available to reach all potentially affected households. This can be achieved by incorporating IK into policy planning and implementation;
- warning systems are inclusive to ensure that nobody is left behind in times of emergencies;
- warning messages integrated with IK should be targeted and focused to specific communities;
- alert messages shoud be integrated with early planning, and inclusion of IK for effective response; and
- there is inter-institutional collaboration and coordination essential for joint actions to build on IK systems at all levels.

### Recommendations

Based on the contribution of IK in DRR and climate change adaptation strategies, our recommendations are as follows:

- Government and development partners should commit to engage researchers to gather and document IK at a wider scale.
- Academicians should do more research on compilation of IK and their roles in DRR and climate change adaptation for the whole country.
- Government through the Ministry of Education should include IK in school curriculum especially in primary and secondary schools to allow the younger generation to understand the importance of IK systems.
- The Department of Climate Change and Meteorological Services should incorporate IK in their messages when providing early warning information to the people at risk.
- Non-governmental organisations supporting vulnerable groups should commit to implement projects related to IK which can help to encourage young people to learn their own local knowledge.
